# Enquiry after Popular Remedies

**Published:** 1808-09

**Authors:** 


*The following important Paper appeared in the last Monthly Magazine, and at the Desire of the Writer, we cheerfully give it a Place in this Journal, and recommend the Object of it to the Attention of all our Readers.*

THE public having experienced the happy effects which have resulted from the adoption of a popular opinion, by Dr. Jenner; and it being a known and obvious Fact that nearly all great discoveries have been the result of accident, of random experience, or of fortuitous observation, I conceive that I shall render an important and acceptable service to mankind, by inviting information relative to all vulgar or popular remedies.

With this view, I formally address myself to the faculty in particular, and to your intelligent, inquisitive, and public-spirited readers of every description; and shall be glad to have accounts of popular opinions and received notions, relative to the origin, cause, prevention, and cure, of diseases, as well in the British islands as in foreign countries; and also of the composition of efficacious family receipts and nostrums, which differ essentially in their ingredients from the prescriptions of the materia medica.

I am perfectly sensible that such remedies and opinions must abound in numerous absurdities, and in gross errors of every kind; yet amidst the mass, I do not fear but I shall find some facts and hints, which, when submitted to the ordeal of regular science, and to the accurate investigation of the faculty, will reward me for my trouble, and prove of the highest service to the human race. I may indeed be able, by these means, to advance the art of physic more within a few months, than it could be improved in a century, without drawing together such an aggregation of experience.

For obvious reasons, I request that every communication may be signed by the name and address of the writer, and authenticated by as many actual references as convenient.

Once in two or three months, with the aid of some medical friends, I will arrange the whole in a form suited to the public eye, and so as to occupy as small a portion of your valuable miscellany as possible. Perhaps, in some cases of consequence, you may be induced hereafter to afford opportunity to your Correspondents to discuss the subjects of communication more at large, and thereby add to the stock of facts which I may be able to accumulate.

For the present, I shall assume the anonymous signature ture by which I have long been known to the readers of your Miscellany, and shall request that all communications may be left for me at your office, No. 6, Bridge-Street, London, free of postage. I am, &c.

COMMON SENSE.

*London, June* 15, 1808.

